# 3-(2,4-Dichloro­phen­yl)-2-oxo-1-oxa­spiro­[4.5]dec-3-en-4-yl 4-chloro­benzoate

**DOI:** 10.1107/S1600536809044109

**Published:** 2009-11-04

**Authors:** Yong Zhou, Jing-Li Cheng, Guo-Nian Zhu, Jin-Hao Zhao

**Affiliations:** a College of Agriculture and Biotechnology, Zhejiang University, Hangzhou 310029, People’s Republic of China

## Abstract

In the title spiro­diclofen derivative, C_22_H_17_Cl_3_O_4_, the cyclo­hexane ring adopts a chair conformation [four C atoms are planar with a mean deviation of 0.018 Å and the two C atoms at the flap positions deviate by 0.613 (4) and −0.668 (5) Å from the plane]. The dihedral angles between the furan ring and the two benzene rings are 55.78 (3) and 49.92 (3)°. Weak inter­molecular C—H⋯Cl inter­actions are observed in the crystal structure.

## Related literature

For the chemistry of tetronic acid, the central unit of the title compound, see: Fischer *et al.* (1993 ▶); Benson *et al.* (2000 ▶). For the pesticides Spiro­diclofen, Spiro­mesifen and Spiro­tetra­mate, see: BAYER Aktiengesellschaft (1995 ▶). For the synthesis and structure of the inter­mediate compound for the preparation of spiro­diclofen, see: Zhao *et al.* (2009 ▶). For the extinction correction, see: Larson (1970 ▶).
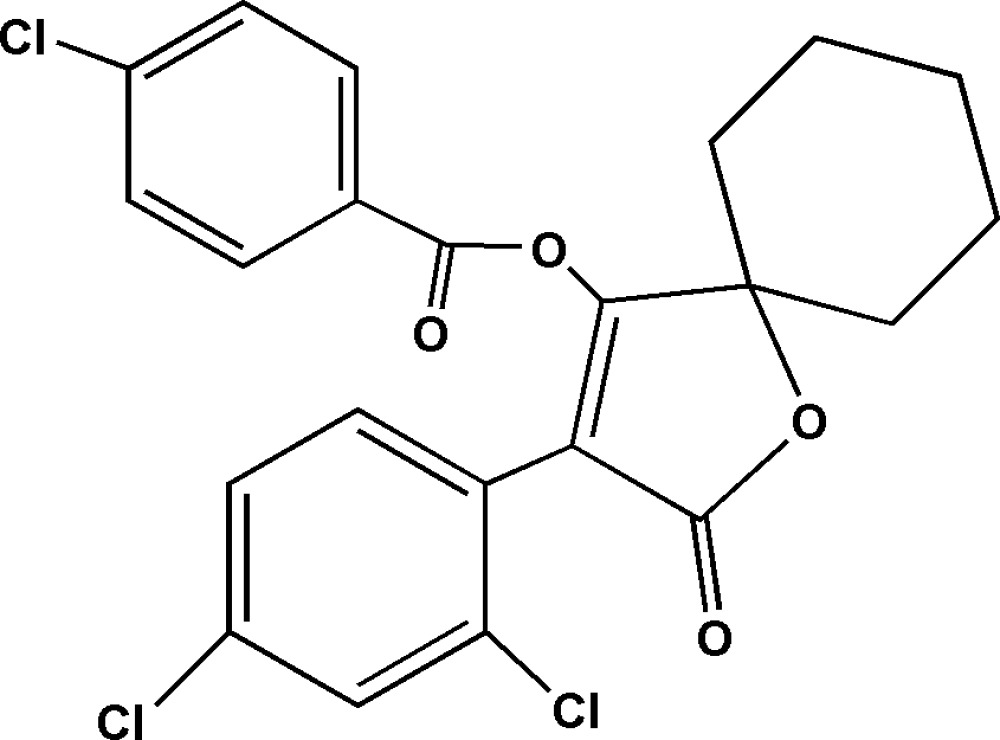


## Experimental

### 

#### Crystal data

C_22_H_17_Cl_3_O_4_
*M* *_r_* = 451.73
Monoclinic, 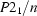

*a* = 14.7979 (8) Å
*b* = 10.3483 (5) Å
*c* = 15.0702 (8) Å
β = 115.1875 (12)°
*V* = 2088.33 (19) Å^3^
*Z* = 4
Mo *K*α radiation
μ = 0.46 mm^−1^
*T* = 296 K
0.46 × 0.32 × 0.28 mm

#### Data collection

Rigaku R-AXIS RAPID diffractometer
Absorption correction: multi-scan (*ABSCOR*; Higashi, 1995 ▶) *T*_min_ = 0.800, *T*_max_ = 0.878
19403 measured reflections
4756 independent reflections
3289 reflections with *F* ^2^ > 2σ(*F* ^2^)
*R*_int_ = 0.029

#### Refinement

*R*[*F* ^2^ > 2σ(*F* ^2^)] = 0.050
*wR*(*F* ^2^) = 0.131
*S* = 1.00
4756 reflections
263 parameters
All H-atom parameters refined
Δρ_max_ = 0.54 e Å^−3^
Δρ_min_ = −0.43 e Å^−3^

### 

Data collection: *PROCESS-AUTO* (Rigaku, 1998 ▶); cell refinement: *PROCESS-AUTO*; data reduction: *CrystalStructure* (Rigaku/MSC, 2002 ▶); program(s) used to solve structure: *SIR97* (Altomare *et al.*, 1993 ▶); program(s) used to refine structure: *CRYSTALS* (Watkin *et al.*, 1996 ▶); molecular graphics: *CRYSTALS*; software used to prepare material for publication: *CrystalStructure* and *PLATON* (Spek, 2009 ▶).

## Supplementary Material

Crystal structure: contains datablocks global, I. DOI: 10.1107/S1600536809044109/si2216sup1.cif

Structure factors: contains datablocks I. DOI: 10.1107/S1600536809044109/si2216Isup2.hkl

Additional supplementary materials:  crystallographic information; 3D view; checkCIF report

## Figures and Tables

**Table 1:** Hydrogen-bond geometry (Å, °)

*D*—H⋯*A*	*D*—H	H⋯*A*	*D*⋯*A*	*D*—H⋯*A*
C5—H52⋯Cl1^i^	0.97	2.79	3.726 (3)	162
C16—H16⋯Cl3^ii^	0.93	2.82	3.595 (3)	141

Symmetry codes: (i) 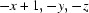; (ii) 
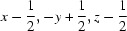
.

**Fig. 1.**
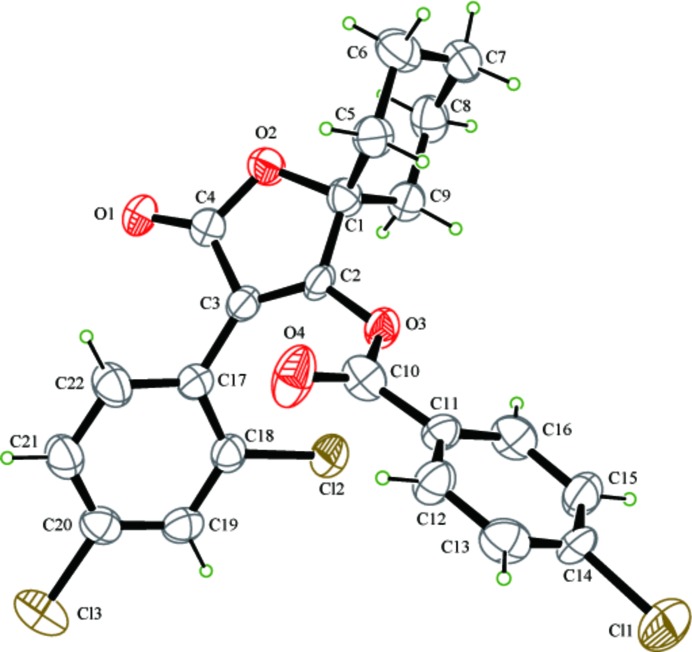
The molecular structure of (I), showing the atom-labelling scheme. Displacement ellipsoids are drawn at the 30% probability level.

**Fig. 2.**
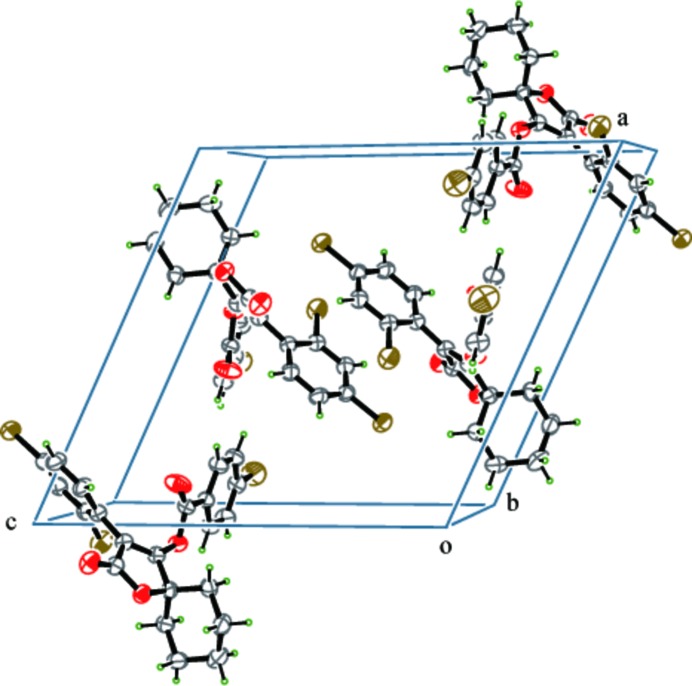
Molecular packing arrangement in the unit cell.

## supplementary crystallographic information

### Comment

The chemistry of tetronic acid compounds has being receiving increasing attention in recent years (Fischer *et al.*,1993; Benson *et al.*, 2000). Bayer company have developed three tetronic acids pesticide-Spirodiclofen, Spiromesifen and Spirotetramat. 4-hydroxyl-3-(2,4-dichlorophenyl)-1- oxaspiro[4,5]dec-3-en-2-one (Zhao *et al.*, 2009) is the key intermediate in preparing highly efficient acaricide-Spirodiclofen (BAYER *et al.*,1995). We have been involved in the synthesis and potential bioactivity of substituted 4-hydroxyl-3-(2,4-dichlorophenyl) -1-oxaspiro[4,5]dec-3-en-2-one. In order to find new compounds which have good bioactivity, we have isolated the product, (I), of the condensation reaction of 4-chlorobenzoyl chloride and 4-hydroxyl-3- (2,4-dichlorophenyl)-1-oxaspiro[4,5]dec-3-en-2-one as colorless crystals suitable for X-ray analysis. The molecular structure of (I) is shown in Fig. 1. The molecule contains three six membered rings and one five membered rings. The dihedral angles between the ring (C11—C16) and ring (C17—C22), ring (C11—C16) and furan ring, ring (C17—C22) and furan ring, are 67.84 (3), 55.78 (3), and 49.92 (3) °, respectively. The cyclohexane ring displays chair conformation with C1 and C7 atoms at the flap position 0.613 (4) and -0.668 (5) Å out of the mean plane formed by the other four atoms. As expected, C2—C3, C4—O1 and C10—O4 are typically double bonds with bond distances of 1.319 (3), 1.199 (3) and 1.190 (3) Å. The bond distance of C3—C4 is 1.487 (3) Å, suggesting that carbonyl group on C7 has formed a conjugate system with double bond on C2 and C3. It is worth notice that in the title compound there exist two weak intermolecular C—H···Cl contacts (Table 1).

### Experimental

4-hydroxyl-3-(2,4-dichlorophenyl)-1-oxaspiro[4,5]dec-3-en-2-one (3.12 g, 10 mmol), 4-dimethylaminopyridine (0.58 g, 1eq.), triethylamine (1.31 g, 1.3eq.) and chloroform (100 ml) were added to a 250 ml round flask. Then the mixture was stirred and cooled to 273–278 K. Within 30 min 4-chlorobenzoyl chloride (1.5eq.) was added dropwise to the solution. The mixture was stirred at room temperature for 3 h and then 1% aqueous HCl was added. The organic layer was washed to neutral with water and dried over Na_2_SO_4_. After filtered and concentrated, the organic residue was purified by silica gel column chromatography, eluted with ethyl acetate-petroleum(1:30,*v*/*v*) to give a white solid (yield 85%, 3.96 g), which was then recrystallized from ethyl acetate/ethanol (1:1,*v*/*v*) to give colourless blocks.

The 1H NMR, EI-MS data testified the title compound's structure. 1H-NMR (500MHz, CDCl3, δ ppm):7.969-7.942 (2H, m, -CO-Ph-H4), 7.492-7.465 (2H, m, -CO-Ph-H4), 7.455-7.301 (3H, m, Cl2-Ph-H3), 1.876-1.802 (10H, m, cyclohexane-H10); EI-MS (70eV, m/z) (relative intensity %): 452 (M+2, 5), 450 (M+, 5),141 (33) ,139 (100). So we are sure that our chemical formula of the compound is correctly presented.

### Refinement

The H atoms were geometrically placed (C–H = 0.93–0.97 Å) and refined as riding with *U*_iso_(H) = 1.2*U*_eq_(C).

### Crystal data

**Table:**

C_22_H_17_Cl_3_O_4_	*F*(000) = 928.00
*M**_r_* = 451.73	*D*_x_ = 1.437 Mg m^−^^3^
Monoclinic, *P*2_1_/*n*	Mo *K*α radiation, λ = 0.71075 Å
Hall symbol: -P 2yn	Cell parameters from 13096 reflections
*a* = 14.7979 (8) Å	θ = 3.0–27.4°
*b* = 10.3483 (5) Å	µ = 0.46 mm^−^^1^
*c* = 15.0702 (8) Å	*T* = 296 K
β = 115.1875 (12)°	Chunk, colorless
*V* = 2088.33 (19) Å^3^	0.46 × 0.32 × 0.28 mm
*Z* = 4	

### Data collection

**Table:**

Rigaku R-AXIS RAPID diffractometer	3289 reflections with *F*^2^ > 2σ(*F*^2^)
Detector resolution: 10.00 pixels mm^-1^	*R*_int_ = 0.029
ω scans	θ_max_ = 27.4°
Absorption correction: multi-scan (*ABSCOR*; Higashi, 1995)	*h* = −19→19
*T*_min_ = 0.800, *T*_max_ = 0.878	*k* = −13→12
19403 measured reflections	*l* = −19→19
4756 independent reflections	

### Refinement

**Table:**

Refinement on *F*^2^	*w* = 1/[0.0005*F*_o_^2^ + 3σ(*F*_o_^2^)]/(4*F*_o_^2^)
*R*[*F*^2^ > 2σ(*F*^2^)] = 0.050	(Δ/σ)_max_ < 0.001
*wR*(*F*^2^) = 0.131	Δρ_max_ = 0.54 e Å^−^^3^
*S* = 1.00	Δρ_min_ = −0.43 e Å^−^^3^
4756 reflections	Extinction correction: Larson (1970), equation 22
263 parameters	Extinction coefficient: 127 (22)
All H-atom parameters refined	

### Special details

**Table:**

Geometry. ENTER SPECIAL DETAILS OF THE MOLECULAR GEOMETRY
Refinement. Refinement using all reflections. The weighted *R*-factor (*wR*) and goodness of fit (*S*) are based on *F*^2^. *R*-factor (gt) are based on *F*. The threshold expression of *F*^2^ > 2.0 σ(*F*^2^) is used only for calculating *R*-factor (gt).

### Fractional atomic coordinates and isotropic or equivalent isotropic displacement parameters (Å^2^)

**Table:**

	*x*	*y*	*z*	*U*_iso_*/*U*_eq_	
Cl1	0.59797 (8)	−0.25919 (9)	0.12627 (8)	0.0964 (3)	
Cl2	0.43688 (6)	0.27225 (6)	0.35188 (6)	0.0706 (2)	
Cl3	0.74849 (6)	0.44566 (10)	0.67014 (5)	0.0783 (2)	
O1	0.41594 (14)	0.74637 (17)	0.27583 (14)	0.0635 (6)	
O2	0.32741 (12)	0.64354 (18)	0.13360 (12)	0.0526 (5)	
O3	0.43940 (12)	0.33403 (16)	0.14982 (12)	0.0499 (5)	
O4	0.60304 (16)	0.3775 (2)	0.2079 (2)	0.0823 (8)	
C1	0.32759 (19)	0.5153 (2)	0.09125 (19)	0.0475 (7)	
C2	0.41627 (18)	0.4558 (2)	0.17081 (19)	0.0426 (7)	
C3	0.45501 (19)	0.5243 (2)	0.25228 (19)	0.0441 (7)	
C4	0.4012 (2)	0.6500 (2)	0.2275 (2)	0.0495 (8)	
C5	0.3374 (2)	0.5343 (2)	−0.00451 (19)	0.0545 (8)	
C6	0.2448 (2)	0.5961 (3)	−0.0843 (2)	0.0693 (10)	
C7	0.1506 (2)	0.5212 (3)	−0.0994 (2)	0.0712 (10)	
C8	0.1395 (2)	0.5091 (3)	−0.0036 (2)	0.0653 (9)	
C9	0.23153 (18)	0.4468 (2)	0.0752 (2)	0.0523 (8)	
C10	0.5376 (2)	0.3002 (3)	0.1748 (2)	0.0548 (9)	
C11	0.54804 (19)	0.1611 (2)	0.15495 (19)	0.0452 (7)	
C12	0.6420 (2)	0.1213 (2)	0.1683 (2)	0.0553 (8)	
C13	0.6566 (2)	−0.0088 (3)	0.1565 (2)	0.0628 (10)	
C14	0.5783 (2)	−0.0928 (2)	0.1331 (2)	0.0552 (8)	
C15	0.4847 (2)	−0.0556 (2)	0.1182 (2)	0.0620 (9)	
C16	0.4699 (2)	0.0755 (2)	0.1285 (2)	0.0586 (9)	
C17	0.53097 (19)	0.5005 (2)	0.35283 (19)	0.0454 (7)	
C18	0.52788 (19)	0.3912 (2)	0.4051 (2)	0.0482 (8)	
C19	0.5946 (2)	0.3713 (2)	0.5033 (2)	0.0528 (8)	
C20	0.6659 (2)	0.4649 (2)	0.5479 (2)	0.0530 (8)	
C21	0.6719 (2)	0.5728 (3)	0.4985 (2)	0.0620 (9)	
C22	0.6042 (2)	0.5900 (3)	0.4017 (2)	0.0620 (9)	
H12	0.6941	0.1801	0.1847	0.066*	
H13	0.7188	−0.0386	0.1644	0.075*	
H15	0.4330	−0.1150	0.1019	0.074*	
H16	0.4067	0.1052	0.1174	0.070*	
H19	0.5908	0.2979	0.5372	0.063*	
H21	0.7210	0.6344	0.5297	0.074*	
H22	0.6084	0.6641	0.3687	0.074*	
H51	0.3944	0.5896	0.0079	0.065*	
H52	0.3481	0.4507	−0.0276	0.065*	
H61	0.2386	0.6841	−0.0654	0.083*	
H62	0.2521	0.5969	−0.1454	0.083*	
H71	0.1544	0.4355	−0.1236	0.085*	
H72	0.0929	0.5662	−0.1472	0.085*	
H81	0.0816	0.4562	−0.0146	0.078*	
H82	0.1306	0.5944	0.0181	0.078*	
H91	0.2245	0.4475	0.1363	0.063*	
H92	0.2356	0.3583	0.0561	0.063*	

### Atomic displacement parameters (Å^2^)

**Table:**

	*U*^11^	*U*^22^	*U*^33^	*U*^12^	*U*^13^	*U*^23^
Cl1	0.1031 (7)	0.0598 (5)	0.1127 (7)	0.0265 (5)	0.0329 (6)	−0.0117 (5)
Cl2	0.0826 (5)	0.0506 (4)	0.0693 (5)	−0.0178 (4)	0.0235 (4)	0.0017 (3)
Cl3	0.0571 (4)	0.1195 (8)	0.0503 (4)	0.0126 (5)	0.0151 (3)	0.0049 (4)
O1	0.0807 (14)	0.0348 (11)	0.0636 (12)	0.0022 (9)	0.0196 (10)	−0.0103 (9)
O2	0.0553 (11)	0.0493 (11)	0.0461 (10)	0.0078 (9)	0.0148 (9)	−0.0039 (9)
O3	0.0434 (10)	0.0383 (10)	0.0612 (11)	0.0034 (8)	0.0158 (8)	−0.0091 (8)
O4	0.0564 (12)	0.0581 (14)	0.138 (2)	−0.0125 (11)	0.0469 (13)	−0.0256 (14)
C1	0.0482 (15)	0.0472 (16)	0.0453 (15)	0.0039 (12)	0.0182 (12)	−0.0063 (13)
C2	0.0475 (14)	0.0247 (13)	0.0544 (16)	0.0009 (11)	0.0206 (13)	−0.0045 (12)
C3	0.0459 (14)	0.0333 (14)	0.0511 (15)	−0.0006 (11)	0.0187 (12)	0.0004 (12)
C4	0.0531 (16)	0.0442 (17)	0.0501 (16)	−0.0031 (13)	0.0208 (14)	−0.0017 (14)
C5	0.0606 (17)	0.0535 (18)	0.0522 (16)	0.0033 (14)	0.0268 (14)	−0.0028 (14)
C6	0.075 (2)	0.081 (2)	0.0465 (16)	0.0048 (18)	0.0204 (15)	0.0007 (16)
C7	0.073 (2)	0.061 (2)	0.0576 (19)	0.0062 (17)	0.0067 (17)	−0.0070 (16)
C8	0.0466 (16)	0.066 (2)	0.075 (2)	0.0016 (14)	0.0172 (15)	−0.0164 (17)
C9	0.0508 (16)	0.0480 (17)	0.0576 (16)	−0.0013 (13)	0.0225 (14)	−0.0034 (14)
C10	0.0412 (15)	0.072 (2)	0.0535 (17)	−0.0007 (15)	0.0229 (13)	−0.0034 (15)
C11	0.0472 (15)	0.0394 (15)	0.0482 (15)	0.0087 (12)	0.0196 (13)	0.0017 (12)
C12	0.0523 (16)	0.0440 (17)	0.0713 (19)	0.0035 (14)	0.0279 (15)	−0.0033 (15)
C13	0.0522 (17)	0.074 (2)	0.0634 (19)	0.0174 (17)	0.0254 (15)	0.0025 (17)
C14	0.071 (2)	0.0293 (14)	0.0596 (18)	0.0143 (14)	0.0227 (16)	−0.0001 (13)
C15	0.0619 (19)	0.0420 (17)	0.077 (2)	0.0028 (15)	0.0241 (16)	−0.0074 (15)
C16	0.0395 (15)	0.072 (2)	0.0635 (18)	0.0048 (15)	0.0213 (14)	−0.0003 (16)
C17	0.0463 (15)	0.0402 (15)	0.0479 (15)	0.0015 (12)	0.0184 (13)	0.0011 (13)
C18	0.0489 (15)	0.0411 (16)	0.0537 (16)	0.0019 (12)	0.0209 (13)	−0.0061 (13)
C19	0.0581 (16)	0.0458 (17)	0.0562 (17)	0.0120 (14)	0.0259 (14)	0.0052 (14)
C20	0.0487 (16)	0.0595 (19)	0.0495 (16)	0.0098 (14)	0.0197 (13)	0.0009 (15)
C21	0.0494 (17)	0.068 (2)	0.0589 (18)	−0.0103 (15)	0.0138 (15)	−0.0052 (16)
C22	0.0545 (17)	0.074 (2)	0.0522 (17)	−0.0163 (15)	0.0176 (15)	−0.0006 (16)

### Geometric parameters (Å, °)

**Table:**

Cl1—C14	1.757 (2)	C15—C16	1.393 (4)
Cl2—C18	1.747 (2)	C17—C18	1.390 (3)
Cl3—C20	1.736 (2)	C17—C22	1.377 (3)
O1—C4	1.199 (3)	C18—C19	1.402 (3)
O2—C1	1.473 (3)	C19—C20	1.378 (3)
O2—C4	1.373 (2)	C20—C21	1.366 (4)
O3—C2	1.377 (3)	C21—C22	1.386 (3)
O3—C10	1.382 (3)	C5—H51	0.970
O4—C10	1.190 (3)	C5—H52	0.970
C1—C2	1.483 (3)	C6—H61	0.970
C1—C5	1.523 (4)	C6—H62	0.970
C1—C9	1.513 (3)	C7—H71	0.970
C2—C3	1.319 (3)	C7—H72	0.970
C3—C4	1.487 (3)	C8—H81	0.970
C3—C17	1.473 (3)	C8—H82	0.970
C5—C6	1.527 (3)	C9—H91	0.970
C6—C7	1.524 (4)	C9—H92	0.970
C7—C8	1.526 (5)	C12—H12	0.930
C8—C9	1.517 (3)	C13—H13	0.930
C10—C11	1.492 (4)	C15—H15	0.930
C11—C12	1.380 (4)	C16—H16	0.930
C11—C16	1.374 (3)	C19—H19	0.930
C12—C13	1.386 (4)	C21—H21	0.930
C13—C14	1.369 (4)	C22—H22	0.930
C14—C15	1.361 (4)		
			
C1—O2—C4	110.07 (18)	Cl3—C20—C21	119.5 (2)
C2—O3—C10	120.29 (19)	C19—C20—C21	121.6 (2)
O2—C1—C2	100.83 (17)	C20—C21—C22	119.5 (2)
O2—C1—C5	108.2 (2)	C17—C22—C21	121.9 (2)
O2—C1—C9	108.5 (2)	C1—C5—H51	108.7
C2—C1—C5	114.3 (2)	C1—C5—H52	108.7
C2—C1—C9	112.5 (2)	C6—C5—H51	108.7
C5—C1—C9	111.7 (2)	C6—C5—H52	108.7
O3—C2—C1	114.5 (2)	H51—C5—H52	109.5
O3—C2—C3	130.8 (2)	C5—C6—H61	109.0
C1—C2—C3	114.5 (2)	C5—C6—H62	109.0
C2—C3—C4	105.2 (2)	C7—C6—H61	109.0
C2—C3—C17	135.0 (2)	C7—C6—H62	109.0
C4—C3—C17	119.8 (2)	H61—C6—H62	109.5
O1—C4—O2	121.7 (2)	C6—C7—H71	109.1
O1—C4—C3	129.5 (2)	C6—C7—H72	109.1
O2—C4—C3	108.8 (2)	C8—C7—H71	109.1
C1—C5—C6	112.7 (2)	C8—C7—H72	109.1
C5—C6—C7	111.2 (2)	H71—C7—H72	109.5
C6—C7—C8	111.2 (2)	C7—C8—H81	109.2
C7—C8—C9	110.7 (2)	C7—C8—H82	109.2
C1—C9—C8	113.3 (2)	C9—C8—H81	109.2
O3—C10—O4	121.2 (2)	C9—C8—H82	109.2
O3—C10—C11	112.1 (2)	H81—C8—H82	109.5
O4—C10—C11	126.7 (2)	C1—C9—H91	108.5
C10—C11—C12	116.3 (2)	C1—C9—H92	108.5
C10—C11—C16	122.3 (2)	C8—C9—H91	108.5
C12—C11—C16	121.4 (2)	C8—C9—H92	108.5
C11—C12—C13	118.2 (2)	H91—C9—H92	109.5
C12—C13—C14	119.3 (3)	C11—C12—H12	120.9
Cl1—C14—C13	119.7 (2)	C13—C12—H12	120.9
Cl1—C14—C15	116.7 (2)	C12—C13—H13	120.4
C13—C14—C15	123.6 (2)	C14—C13—H13	120.4
C14—C15—C16	117.0 (2)	C14—C15—H15	121.5
C11—C16—C15	120.5 (2)	C16—C15—H15	121.5
C3—C17—C18	121.6 (2)	C11—C16—H16	119.8
C3—C17—C22	121.3 (2)	C15—C16—H16	119.8
C18—C17—C22	116.9 (2)	C18—C19—H19	121.3
Cl2—C18—C17	121.20 (17)	C20—C19—H19	121.3
Cl2—C18—C19	116.1 (2)	C20—C21—H21	120.2
C17—C18—C19	122.6 (2)	C22—C21—H21	120.2
C18—C19—C20	117.4 (2)	C17—C22—H22	119.1
Cl3—C20—C19	119.0 (2)	C21—C22—H22	119.1
			
C1—O2—C4—O1	178.8 (3)	C17—C3—C4—O2	−173.4 (2)
C1—O2—C4—C3	0.2 (3)	C1—C5—C6—C7	−53.4 (3)
C4—O2—C1—C2	−4.5 (3)	C5—C6—C7—C8	55.8 (3)
C4—O2—C1—C5	−124.7 (2)	C6—C7—C8—C9	−56.1 (3)
C4—O2—C1—C9	113.9 (2)	C7—C8—C9—C1	54.5 (3)
C2—O3—C10—O4	−5.7 (4)	O3—C10—C11—C12	173.7 (2)
C2—O3—C10—C11	174.4 (2)	O3—C10—C11—C16	−8.8 (4)
C10—O3—C2—C1	137.8 (2)	O4—C10—C11—C12	−6.2 (5)
C10—O3—C2—C3	−48.0 (4)	O4—C10—C11—C16	171.4 (3)
O2—C1—C2—O3	−176.7 (2)	C10—C11—C12—C13	176.1 (2)
O2—C1—C2—C3	8.1 (3)	C10—C11—C16—C15	−174.9 (2)
O2—C1—C5—C6	−68.4 (2)	C12—C11—C16—C15	2.6 (4)
O2—C1—C9—C8	67.4 (3)	C16—C11—C12—C13	−1.5 (4)
C2—C1—C5—C6	−179.8 (2)	C11—C12—C13—C14	−0.5 (4)
C5—C1—C2—O3	−60.9 (3)	C12—C13—C14—Cl1	−175.6 (2)
C5—C1—C2—C3	124.0 (2)	C12—C13—C14—C15	1.5 (4)
C2—C1—C9—C8	178.0 (2)	Cl1—C14—C15—C16	176.8 (2)
C9—C1—C2—O3	67.9 (3)	C13—C14—C15—C16	−0.4 (4)
C9—C1—C2—C3	−107.2 (3)	C14—C15—C16—C11	−1.6 (4)
C5—C1—C9—C8	−51.8 (3)	C3—C17—C18—Cl2	3.1 (4)
C9—C1—C5—C6	51.0 (3)	C3—C17—C18—C19	−174.9 (3)
O3—C2—C3—C4	177.6 (3)	C3—C17—C22—C21	175.5 (3)
O3—C2—C3—C17	−4.5 (6)	C18—C17—C22—C21	−0.2 (4)
C1—C2—C3—C4	−8.2 (3)	C22—C17—C18—Cl2	178.7 (2)
C1—C2—C3—C17	169.7 (3)	C22—C17—C18—C19	0.8 (4)
C2—C3—C4—O1	−173.7 (3)	Cl2—C18—C19—C20	−178.7 (2)
C2—C3—C4—O2	4.9 (3)	C17—C18—C19—C20	−0.6 (4)
C2—C3—C17—C18	−50.1 (5)	C18—C19—C20—Cl3	178.8 (2)
C2—C3—C17—C22	134.5 (3)	C18—C19—C20—C21	−0.1 (4)
C4—C3—C17—C18	127.6 (3)	Cl3—C20—C21—C22	−178.2 (2)
C4—C3—C17—C22	−47.9 (4)	C19—C20—C21—C22	0.7 (5)
C17—C3—C4—O1	8.0 (5)	C20—C21—C22—C17	−0.6 (5)

### Hydrogen-bond geometry (Å, °)

**Table:**

*D*—H···*A*	*D*—H	H···*A*	*D*···*A*	*D*—H···*A*
C5—H52···Cl1^i^	0.97	2.79	3.726 (3)	162
C16—H16···Cl3^ii^	0.93	2.82	3.595 (3)	141

Symmetry codes: (i) −*x*+1, −*y*, −*z*; (ii) *x*−1/2, −*y*+1/2, *z*−1/2.
